# Procrastination

**Published:** 1889-02

**Authors:** 


### PROCRASTINATION.

"Why don't you mend your roof, my man ?"
He drew him closer to the wall,
And answered with a lazy drawl :
"When 't rains so hard I never can."

"Why don't you mend it when its fair ?"
"O then it doesn't need repair !"
He blandly said
As he turned his head
And shook the raindrops from his hair.

So it is with the sick who wait
The means of cure that come too late.

